# Lack of orientation specific adaptation to vertically oriented Glass patterns in human visual cortex: an fMRI adaptation investigation

**DOI:** 10.1038/s41598-023-39247-7

**Published:** 2023-07-31

**Authors:** Andrea Pavan, Wilhelm M. Malloni, Sebastian M. Frank, Simon Wein, Rita Donato, Mark W. Greenlee

**Affiliations:** 1 Department of Psychology, University of Bologna, Viale Berti Pichat, 5, 40127 Bologna, Italy; 2 Institute for Experimental Psychology, University of Regensburg, 93053 Regensburg, Germany; 3 School of Psychology, University of Lincoln, Brayford Pool, Lincoln, LN6 7TS UK; 4 Department of General Psychology, University of Padova, Padova, Italy; 5 Proaction Laboratory, Faculty of Psychology and Educational Sciences, University of Coimbra, Colégio de Jesus, Rua Inácio Duarte 65, 3000-481 Coimbra, Portugal; 6 CINEICC, Faculty of Psychology and Educational Sciences, University of Coimbra, Rua Colégio Novo, 3000-115 Coimbra, Portugal

**Keywords:** Human behaviour, Visual system

## Abstract

The perception of coherent form configurations in natural scenes relies on the activity of early visual areas that respond to local orientation cues. Subsequently, high-level visual areas pool these local signals to construct a global representation of the initial visual input. However, it is still debated whether neurons in the early visual cortex respond also to global form features. Glass patterns (GPs) are visual stimuli employed to investigate local and global form processing and consist of randomly distributed dots pairs called dipoles arranged to form specific global configurations. In the current study, we used GPs and functional magnetic resonance imaging (fMRI) adaptation to reveal the visual areas that subserve the processing of oriented GPs. Specifically, we adapted participants to vertically oriented GP, then we presented test GPs having either the same or different orientations with respect to the adapting GP. We hypothesized that if local form features are processed exclusively by early visual areas and global form by higher-order visual areas, then the effect of visual adaptation should be more pronounced in higher tier visual areas as it requires global processing of the pattern. Contrary to this expectation, our results revealed that adaptation to GPs is robust in early visual areas (V1, V2, and V3), but not in higher tier visual areas (V3AB and V4v), suggesting that form cues in oriented GPs are primarily derived from local-processing mechanisms that originate in V1. Finally, adaptation to vertically oriented GPs causes a modification in the BOLD response within early visual areas, regardless of the relative orientations of the adapting and test stimuli, indicating a lack of orientation selectivity.

## Introduction

There is psychophysical, neuroimaging, and computational evidence attesting to the notion that perception of coherent structures in natural images relies on the activity of early visual detectors such as in V1/V2 that respond to local orientation signals, whereas higher-order visual areas such as inferotemporal (IT) visual regions pool local information to form the representation of complex objects^1–7^. Previous behavioral research has extensively used Glass patterns (GPs)^8^ to investigate how complex visual scenes can be derived from local orientation signals^4,7,9–11^, however, the neural basis that drives this process is still debated^4,5,12–15^. GPs are textures formed by multiple dot pairs, called dipoles, spatially arranged according to specific geometric transformations to create different simple and complex global configurations^16,17^. For these features, GPs have been broadly used to investigate the pooling of local orientation signals into global coherent percepts^18–22^. Early research by Dakin^19^ proposed a general two-stage model for how global orientation information is extracted from GPs. According to this two-stage model, the local orientation of the dipoles that form a GP stimulates the receptive fields of neurons in different cortical columns. This causes an intracolumnar excitation that leads to the pooling of the local orientation signals in the second stage. This model has been supported by computational modelling and studies on spatial filtering^23–26^. Whilst the two-stage model provides an outline for how a global pattern could be perceived, it does not identify the stage at which GP processing occurs. Several neuroimaging and brain stimulation studies have sought to shed light on this mechanism and have shown that oriented GPs are processed not only by the primary visual cortex (area V1) but also by extrastriate areas such as V2, V3, V4, and the lateral occipital complex (LOC)^4^. Consistent with these findings, Swettenham et al.^14^ using magnetoencephalography (MEG) assessed the neural activity associated with the perception of global form from different types of GPs (i.e. horizontal, circular, and radial). To minimize the neural activity in response to low-level properties of the stimulus and to assess the importance of area V1 in global form processing, the local features of the patterns (e.g. contrast and luminance) were held constant throughout the MEG sessions. The results showed that the location of greatest power change was near or within visual area V3A, but no peaks of activity were observed in V1. Additionally, a time–frequency analysis indicated that the neural activity was lower for horizontal patterns than for more complex shapes indicating that participants were less sensitive to horizontal GPs than to circular and radial GPs. This evidence suggests that extrastriate areas may be involved in the pooling of local orientation signals, and consequently in the perception of global form from GPs. However, two questions remain unsolved: firstly, whether horizontal GPs are more difficult to perceive than circular and radial GPs because they may rely on local summation processing rather than global summation mechanisms^7^; secondly, whether the same pattern of results can be obtained with other simple configurations of GPs such as vertical GPs. This question arises because there is evidence that vertical GPs, either dynamic or static, are perceived more easily than horizontal GPs, the so-called “horizontal effect”^27^ (see Donato et al.^28^ for a review). This effect was previously studied in natural images by Hansen and Essock^29^, who showed that in the external environment, individuals are more used to seeing a vertical organization of the visual elements instead of a horizontal structure.

While it is possible that oriented GPs are processed according to the two-stage model, neuroimaging and brain stimulation research has provided evidence that vertically oriented GPs may also be processed at low-level stages of visual analysis^4,15^. For example, Ostwald et al.^4^ using functional magnetic resonance imaging (fMRI) in healthy participants, showed that visual information in GPs is integrated according to a continuum that extends from the processing of local orientation information in early visual areas to the processing of global form information in higher occipitotemporal areas. Using multivoxel pattern analysis (MVPA) the authors found that higher-order occipitotemporal areas code differences in global form, rather than low-level stimulus properties, with these higher visual areas exhibiting greater accuracy than early visual areas, consistent with the hypothesis of global pooling mechanisms of local orientation signals. In addition, classification accuracy in early visual areas (e.g. V1 and V2) was similar for all the GPs used (radial, concentric, and vertically oriented), even though the lateral occipital complex (LOC) exhibited higher classification accuracy for all the presented visual stimuli. Furthermore, Aspell et al.^30^ using fMRI, found that simple orientations such as horizontal and vertical line segments activate various visual areas beyond V1, and that intermediate retinotopic areas V2 and V3 could differentiate vertical and horizontal forms. These results suggest that the processing of vertically oriented GPs may occur mainly at low stages of visual processing, but it does not exclude the contribution of higher-order visual areas. Nevertheless, brain imaging studies reported that concentric, radial, and polar GPs induce greater activation in visual areas such as V3 and V4^31,32^. Taken together these results indicate that global integration of spatial cues with multiple orientation signals is processed by neurons that have larger receptive fields than those in the early stage of visual processing, and they exhibit a higher level of complexity^33,34^. Nevertheless, the findings from neuroimaging and repetitive transcranial magnetic stimulation (rTMS) studies challenge this notion in the context of vertically oriented GPs^4,15^. Hence, additional investigations are warranted to gain better a deeper understanding of the specific stage within the visual system where local and global processing occur in relation to vertically oriented GPs. There is evidence based on brain stimulation that when rTMS is delivered over the human early visual areas V1/V2, the discrimination of static and dynamic (20 Hz) translational GPs is impaired with respect to a noisy GP, i.e. where dipoles were randomly oriented^15^. This suggests not only the fundamental involvement of low-level visual areas but also that the temporary disruption of V1/V2 activity prevents the forwarding of visual information to higher-level visual areas. Besides, there is physiological evidence in macaque monkeys that simple and complex brain cells in early visual areas V1/V2 show selectivity for orientation cues contained in static vertically oriented GPs presented in their classical receptive field^6,12^. Further elucidating the function of early and higher visual areas in form processing at various spatial and temporal characteristics, Kourtzi and Huberle^35^ used fMRI and a series of Gabor elements as visual stimuli. Participants were involved in a dual task—firstly, they had to identify whether the global contours of the two stimuli displayed were the same or different, secondly, they had to indicate whether the local orientation of the set of Gabor forming the contour was the same or different. The results revealed significant activation of the LOC for processing contours, but only weak activation of the early visual areas. In contrast, the early visual regions displayed a high activation relative to changes in the Gabors’ local orientation.

Further results have been reported by Mannion et al.^32^ who investigated how the human visual cortex represented the orientation structure of spatial forms in vertically oriented and polar GPs. They evaluated blood oxygenation level-dependent (BOLD) responses from the early retinotopic areas V1, V2, V3, V3AB, and hV4 using fMRI. V1 exhibited a high sensitivity to vertically oriented GPs, whereas all other visual regions showed a high sensitivity for dipole orientations that were radially arranged with respect to the fixation point. Nonetheless, V1, V2, V3, and hV4 also showed a bias towards dipoles oriented tangentially to the fixation point which was exclusively observed for polar GPs. This enhanced activation to tangential orientations within polar form suggests that early visual areas are sensitive to either curvature or global form features. This observation might indicate that although a considerable amount of evidence indicates a difference in local and global form processing, other studies question this dichotomy pointing to some neural populations in early visual cortical areas that also respond to global form features^36–38^. For example, in a previous study, we showed that adaptation to oriented GPs (i.e. ± 15° from vertical) produces a tilt after-effect (TAE); that is, after prolonged inspection of an oriented pattern, a subsequently presented oriented pattern is perceived as tilted opposite to the orientation of the adapting pattern^39,40^. In this case, the TAE from adaptation to oriented GPs is likely to rely on visual processing levels in which the global orientation of GPs has been encoded by orientation-selective neurons^21,41^. In another study, we showed that GP-induced TAE exhibits an almost complete interocular transfer (i.e. when adaptation to one eye transfers to the other non-adapted eye), indicating the involvement of orientation selective and binocularly driven neurons^21^. In summary, our previous behavioral findings suggest that the TAE from GPs possibly relies on visual processing levels in which the global orientation of GPs is encoded by neurons that are orientation selective, mostly binocularly driven, and sensitive to high temporal frequencies.

These effects are consistent with form-motion integration at low and intermediate levels of visual processing^21,41^. Finally, other studies used fMRI adaptation to gauge orientation selectivity in the human visual cortex and found that not only the early visual areas V1, V2, and V3 but also the higher tier visual areas are orientation-selective to orientation-specific adaptation^42–45^. For instance, Montaser-Kouhsari et al.^44^ measured orientation-selective responses in the human visual cortex to illusory contours, composed of adjacent and phase-shifted lines. They found orientation-selective adaptation to illusory contours in early visual areas such as V1/V2 but also in high-level visual areas such as V3, V4, VO1, V3A/B, V7, LO1 and LO2.

In the present study, using the fMRI adaptation paradigm, we examined the selectivity for global orientation in the human visual system. The proposed research aims to investigate the neural processes that integrate local orientation signals in early visual areas to produce selectivity for global orientation in higher occipitotemporal areas. Our study contributes to our understanding of how form cues are processed in oriented GPs and the visual areas involved. To this aim, we will use a top-up adaptation protocol^44^ to look for orientation-selective neural responses to vertically oriented GPs in the human visual cortex. We measured fMRI responses to GPs of different orientations after adapting to a vertically oriented GP. We decided to adapt participants to vertically oriented GPs because, as previously mentioned, there is evidence showing that this global configuration is easier to detect than other simple configurations such as horizontal GPs^27^. Moreover, in the current study, fMRI adaptation allowed us to measure orientation-selective responses to GPs in the human visual cortex while observers performed a demanding foveal task to control for spatial attention across conditions^42,44,46–50^. In line with the above-mentioned research, we expected to find significant orientation-selective adaptation in both early visual areas and higher tier visual regions yet with a greater impact over the extrastriate visual areas. In contrast to our initial predictions, our findings suggest that adaptation to oriented GPs takes place in early (V1, V2, and V3) but to a lesser extent in higher tier visual areas (V3AB, V4v), implying that the proportion of neurons selective to oriented GPs is larger in V1, V2, and V3 than in higher tier visual areas. Additionally, we did not find evidence of orientation-tuned adaptation effects in either early visual areas or in higher tier visual areas.

## Methods

### Participants

One of the authors (S.M.F.) and nine participants with normal or corrected-to-normal vision took part in the experiment. MR-compatible myopic or hyperopic corrections (Cambridge Research Systems, Ltd) were worn during fMRI scanning. Participants were naïve to the purposes of the study but all of them had previous experience with fMRI experiments. The number of participants has been estimated using the G*Power software^51^. Based on the study of Weigelt et al.^52^, in their four-way within-subjects ANOVA including as factors *design* (pre-adaptation, top-up, and random orientation adaptation), *area* (V1, V2, and V3), *BOLD peak* (first peak and second peak), and *orientation* (same and different) indicated a *partial-η*^2^ = 0.84 for the main effect of the *area* with 10 participants. This corresponds to an effect size *f* = 2.29 with a power = 0.99. A sensitivity analysis using G*Power showed that specifying the effect size as in SPSS, at an alpha error probability of 0.05, assuming a power of 0.99, a total sample size of 10, and for 20 measurement levels (i.e. five ROIs x four test GPs), would require at least an effect size of *f* = 0.501. Methods conform to the World Medical Association Declaration of Helsinki^53^ and the study has been approved by the Ethics Committee of the University of Regensburg (protocol number: 13-101-0029). Written informed consent was obtained from each participant prior to enrolment in the study.

### Visual apparatus

Stimuli were generated using MATLAB PsychToolbox^54–56^ and were back-projected using a PROPixx projector (VPixx Technologies, Saint-Bruno-de-Montarville, Canada) (refresh rate 60 Hz) onto a translucent screen placed inside the MR scanner bore. The screen had a resolution of 1024 × 768 pixels. Participants viewed the stimuli through a mirror located above their eyes. The distance between the mirror and the translucent screen was 95 cm. The mean luminance of the screen was 86 cd/m^2^, whereas the minimum and maximum luminance values were 0.2 cd/m^2^ and 172 cd/m^2^, respectively. A gamma-corrected lookup table was used so that luminance was a linear function of the digital representation of the image. Observers were in a darkened, RF-shielded MR chamber.

### Stimuli

**Figure 1:**
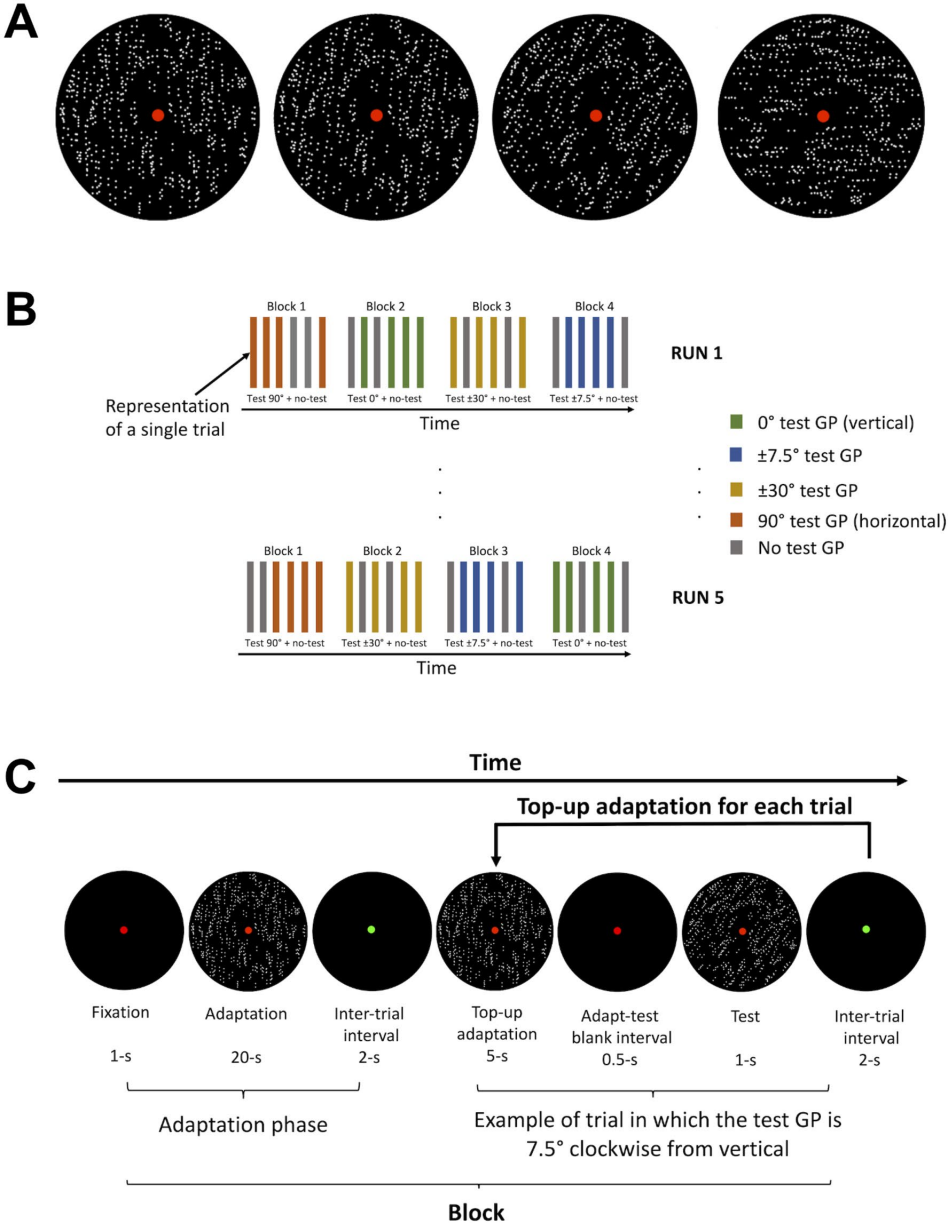
(**A**) Representation of the stimuli used. The Glass patterns (GPs) represented have 100% coherence (i.e. all dipoles are oriented according to the main orientation axis). From left to right: vertically oriented GP (orientation 0°). Vertically oriented GPs were used as adapting and test GPs, test GP oriented at 7.5°, test GP oriented at 30°, and test GP oriented at 90° (horizontal). The second and third GPs are tilted clockwise from vertical. (**B**) Schematic representation of the fMRI block design. (**C**) Representation of one block. See text for more details. For demonstrative purposes, the density of all the GPs represented has been reduced and the dot size increased.

Stimuli were oriented GPs (Fig. 1A) consisting of 2000 dipoles, with each dot having a width of 0.04 deg and an inter-dot spacing of 0.18 deg^18^. Michelson contrast of the dots was c = 0.99, calculated by determining the difference between the dot and background luminance and dividing these by the sum of the two luminance values. The GPs were displayed within an annulus with an outer radius of ~ 6.5 deg and an inner radius of ~ 1 deg. The density was approximately 15.4 dipoles/deg^2^. To avoid retinal afterimages following the prolonged inspection of the patterns and to strengthen orientation adaptation, adapting and test GPs were dynamic. To produce tight packing, a slight overlap of the dipoles was allowed^22^ (Fig. 1A). Dynamic GPs were created by displaying a series of stationary GPs in temporal sequence at 2.0 Hz. That is, for each new presentation (duration: 0.5 s) a new spatial arrangement of the dipoles was created, while their orientation remained constant. This produced a texture updated at 2.0 Hz in which non-directional apparent motion was perceived along the axis parallel to the dipoles’ orientation, though there was no dipole-to-dipole correspondence between successive frames^13,27,41,57–60^.

Adapting GPs were always vertically oriented, whereas test GPs were oriented according to six orientation contrasts with respect to the adapting GP: 0° (i.e. vertical, and same orientation to the adapting GP),  ± 7.5°, ± 30°, 90° (horizontal)^45^. Additionally, GPs were always presented at maximum coherence; that is, all dipoles were oriented according to the main orientation axis (i.e. either 0°, ± 7.5°, ± 30° or 90°).

### Procedure

An fMRI adaptation technique with a *block design* was used. The fMRI adaptation sequence consisted of 5 runs and each run consisted of 4 blocks (Fig. 1B). Each block consisted of an initial fixation of 1 s, an adaptation phase to a vertically oriented GP of 20 s, an inter-trial interval of 2 s, and then a sequence of 6 trials. The duration of each trial was ~ 6.5 s, the duration of each block was ~ 85 s, the duration of each run was ~ 5.67 min and the total duration of the experimental session was approximately 28.3 min. Between each block and each run, there was a blank interval of 11 s, in which there was no task at fixation.

In each trial, participants were adapted to a vertically oriented GP for 5 s (i.e. top-up adaptation). After the top-up adaptation, there was a blank interval of 0.5 s and then a test GP was presented for 1 s. The adapting GPs (adapt and top-up) were always vertical and with a coherence of 100% (i.e. all dipoles were vertically oriented). Likewise, the orientation of the test GP was constant within each block and could be either vertical (0°), 7.5° clockwise or counterclockwise from vertical, 30° clockwise or counterclockwise from vertical, or horizontal (90°).

In this study, we did not test whether the adaptation to vertically oriented GPs influenced the perceived tilt of the test GPs. Under very similar conditions, adaptation to vertically oriented GPs or gratings leads observers to report a weak or no orientation bias in the subsequently presented test stimulus^21,61,62^. The purpose was to measure the magnitude of net neural adaptation to an oriented texture that induces scarce or no perceptual bias onto a subsequently presented oriented stimulus. However, even considering the optimal orientation adaptation that produces the peak tilt after-effect (TAE) (usually around 15°), in the case of oriented (static) GPs, the TAE magnitude is relatively small (between 1.7 and 2.0 deg; see Pavan et al.^21,41^).

For each block, in four trials the test GP was presented, whereas in the remaining two trials no test GP was presented (i.e. blank display). This is important to assess the visual areas in which adaptation to vertically oriented GPs takes place. The order of trials with and without test GP was randomized for each block. The fMRI design and block sequence are represented in Fig. 1B and C, respectively.

Throughout the experiment, and more specifically within each block, participants were required to perform a demanding attentional task at fixation. During the adaptation phase and during each trial, the red fixation point at the center of the screen increased in size for ~ 17 ms (from 0.15 to 0.21 deg). The participants had to count the number of size changes that occurred in each block. Responses were recorded via a fiber-optic response box. The minimum number of fixation-point changes was 1 and the maximum was 4. Participants responded during the inter-trial intervals of 2 s when the fixation point turned green (see Fig. 1C). The task at fixation was necessary because spatial attention can modulate neuronal responses to visual stimuli measured with fMRI in a spatially specific manner^46,47,63–66^. Furthermore, attention modulates aftereffects including the motion aftereffect^67–69^, and tilt aftereffect from illusory contours, gratings, and vertically oriented GPs^21,44,58,70^.

### Data acquisition

MR images were obtained using a 3.0 Tesla Siemens MAGNETOM Prisma system using a 64-channel head/neck coil. A single-shot gradient-echo echoplanar imaging (EPI) was used to acquire BOLD functional images (TR = 1000 ms, TE = 30 ms, flip angle = 52°, slice gap = 0.2 mm, FOV = 192 × 192 mm^2^, dimension = 104 × 104 × 72, voxel size = 2 × 2 × 2 mm^3^). In each image volume, 72 axial slices were acquired using an ascending interleaved scanning sequence with a multi-band (MB) acceleration factor = 6^71^. Additionally, a high-resolution T1-weighted image was acquired. We used a modified version of the MP-RAGE (3D “magnetization prepared rapid gradient echo”) sequence from “The Alzheimer’s Disease Neuroimaging Initiative”^72^. We obtained 208 slices with a resolution of 0.8 × 0.8 × 0.8 mm^3^ using a FOV = 256 × 256 mm^2^. The TR was 2400 ms, the TE 2.18 ms and the flip angle 8°. Functional T2*weighted images were acquired before the structural images. Regions-of-interest (ROIs) were determined using retinotopic mapping techniques.

### Retinotopic mapping

Retinotopically organized areas in the visual cortex (V1, V2, V3, V3AB, and V4v) were localized by means of phase-encoded retinotopic mapping^73–75^. This localizer was collected in our participants for the purpose of another study (see Frank et al.^76^). For phase-encoded retinotopic mapping a bowtie-shaped double-wedge checkerboard pattern flickering in different colors (flicker frequency = 8 Hz) rotated clockwise and counter-clockwise directions across different retinotopic locations (18 locations in total, 3 s for each location, total duration of one rotation cycle = 54 s). There was a total of 12 cycles for each of the clockwise and counterclockwise rotation directions. Clockwise and counterclockwise rotations were conducted in separate runs and there was one run for each direction of rotation (run duration = 10.8 min). During the localizer scans, participants maintained their fixation on a central dot and performed a speeded dimming-detection task on the fixation dot.

Functional imaging data were collected with the above-described 3.0 Tesla Siemens Prisma MRI scanner using the same 64-channel head/neck coil and an echoplanar imaging sequence (TR = 1 s, TE = 33 ms, multiband factor 4, flip angle (FA) = 59°, in-plane acquisition matrix (AM) = 96 × 96, 48 axial slices, voxel size 2.5 × 2.5 × 2.5 mm, no interslice gap). For each participant, a high-resolution T1-weighted anatomical scan of the brain was collected using an MPRAGE sequence (TR = 2.3 s, TE = 2.32 ms, FA = 8°, AM = 256 × 256, 192 sagittal slices, voxel size = 0.9 × 0.9 × 0.9 mm, interslice gap = 0.45 mm). Each participant’s high-resolution anatomical scan of the brain was reconstructed and inflated using the FreeSurfer software package^77,78^ (https://surfer.nmr.mgh.harvard.edu/; Martinos Center for Biomedical Imaging). Phase-encoded retinotopic mapping data were pre-processed (including motion-correction, co-registration to the reconstructed high-resolution anatomical brain, smoothing with a 3D Gaussian kernel [FWHM = 3 mm], intensity-normalization) and analyzed using FreeSurfer’s FSFast toolbox. Visual areas were defined at a threshold of *p* < 0.001 (FDR correction) on each participant’s inflated cortical surfaces.

### Data pre-processing

Following functional image reconstruction, the Statistical Parametric Mapping software package (SPM12, Wellcome Department of Cognitive Neurology, London, UK) was used to perform motion correction by realigning the functional images to the first volume in each run. The displacement parameters in the x, y, and z directions were recorded and used to assess head motion. The maximum net displacement was calculated as the norm of the vector determined by the maximum absolute displacement in each direction. All volumes were realigned spatially to the first volume and the time-series for voxels within each slice were temporally realigned to the middle slice. The resulting volumes were normalized to a standard EPI template based on the MNI reference brain in Talairach space^79^ and resampled to 2 × 2 × 2 mm^3^ voxels. Head movements did not exceed ± 2 mm in any direction during a session. Successively, normalized images were used. The time-series in each voxel were high-pass-filtered with a cutoff frequency of 0.008 Hz to remove low-frequency signal drifts. The structural T1-weighted image was co-registered to the mean functional image of the corresponding run for subsequent display of functional activations.

### fMRI data analysis

We performed the first-level single-subject analysis based on the general linear model (GLM)^80^ with eight regressors (fixation, adaptation, top-up adaptation, four test orientations, and an additional blank/no-test). Six additional covariates (regressors) were included to capture residual movement-related artifacts (the three rigid-body translations and rotations determined from the realignment stage) and a single covariate representing the mean (constant) over scans. For the fast event-related design, the basis function consisted of the canonical hemodynamic response function (*HRF*) model. The statistical maps generated from each task were designed with a threshold at *p* < 0.05 using a family-wise error (FWE) correction^81^ for multiple comparisons. We also performed a random-effects paired *t test* spatially normalized to the Montreal Neurological Institute (MNI) space. Successively, we performed the second-level analysis for analyzing whole-brain group effects of different stimulus orientations.

Analyses were performed on the relative BOLD signal change values obtained by subtracting the fitted BOLD signal change measured in the blank condition from that of each of the test conditions (i.e. 0°, 7.5°, 30° and 90°) (see Supplementary Material for more details about the raw data). This operation was performed individually for each participant. To assess the differences between the relative BOLD signal change values for each test condition within each ROI (i.e. the stimulus-evoked response), relative BOLD signal change values were again fitted with a canonical hemodynamic response function^82,83^.

In a subsequent analysis, we computed an adaptation index (AI) for each visual area to assess and quantify the difference between fMRI responses to the different test GP orientations after vertical adaptation in each visual area. The adaptation index was calculated as in Montaser-Kouhsari et al.^44^:

1 $$AI=\frac{{A}_{\perp }-{A}_{\theta }}{\left|{A}_{\perp }\right|+\left|{A}_{\theta }\right|},$$

where ${A}_{\theta }$ is the mean response amplitude to the different test GPs orientations (i.e. 0°, 7.5°, and 30°), whereas ${A}_{\perp }$ is the mean response amplitude relative to the orthogonal test (i.e. test orientation at 90°). The mean response amplitude is defined as the BOLD signal change estimated in the blank test condition subtracted from the BOLD response amplitude of the oriented test patterns. In this case, the AI reflects the magnitude of vertical adaptation on the presentation of the three absolute test GP orientations (i.e. 0°, 7.5°, and 30°). The index ranges from − 1 to + 1 and is + 1 if adaptation is maximally effective by reducing the response to the test stimulus to zero. Otherwise, if adaptation is not effective, i.e. responses to the different test GP orientations were the same and not affected by adaptation, the index is equal to zero. The index is negative when the adaptation has a facilitatory effect on the subsequently presented test pattern or when the response to the orthogonal test is zero.

## Results

### Behavioral responses to the fixation task

For the attention task during each block, we calculated correct performance for each adaptation type (i.e. adaptation and top-up adaptation), dependent on whether there was a temporal overlap between the size change of the fixation point and a specific phase of the trial. The overall accuracy at the fixation task was 61% (SD: 17.6%). A one-sided one-sample permutation test was performed to assess whether the accuracy for the fixation task was greater than the chance level (0.25). The chance level was set at 0.25 because the maximum number of fixation-point changes was four and participants, on each trial, could select one amongst four possible responses. The test revealed that the accuracy was significantly higher than the chance level (*t* = 6.48, *p* < 0.001), though the task at fixation was apparently quite demanding as participants were not at ceiling. In fact, the same test revealed that the accuracy was significantly lower than 1.0 (i.e. ceiling performance, *t* = − 6.97, *p* < 0.001).

### Results for test GP orientations within each ROI

To assess for differences in response amplitudes across visual areas and test orientations, relative BOLD signal change amplitude values were analyzed with a linear mixed model including as fixed effects the ROI and the test orientation, and as random effect the intercept across participants. According to a Shapiro–Wilk test, the residuals were not normally distributed (*W* = 0.948, *p* < 0.001), with a moderate skewness of 0.715 (SE: 0.172). Using the median absolute deviation with a cut-off of 3^84^, we also identified 20 outliers that were nevertheless included in the analysis. Therefore, we used the Aligned Rank Transform (ART), a procedure for the non-parametric analysis of variance in multifactor designs^85–87^. The analysis was performed in R (v4.2.2) (https://www.r-project.org)^88^. With this technique, a linear mixed model can be implemented once the data are aligned and ranked for each main and interaction effect. Pairwise comparisons were conducted using the ART-C procedure^89^. A linear mixed model with random intercept across participants and including ROI and test orientation as within-subjects factors revealed only a significant effect of the ROI (*F*_4, 171_ = 47.4, *p* < 0.0001) but no effect of test orientation (test orientation: *F*_3, 171_ = 1.789, *p* = 0.151; interaction between ROI and test orientation: *F*_12, 171_ = 0.082, *p* = 0.999). FDR-corrected post hoc comparisons for the ROI (correction for 10 tests) showed a significant difference between: V1 and V3 (*p*_*adj*_ < 0.0001), V1 and V3AB (*p*_*adj*_ < 0.0001), V1 and V4v (*p*_*adj*_ < 0.0001), V2 and V3 (*p*_*adj*_ < 0.0001), V2 and V3AB (*p*_*adj*_ < 0.0001), V2 and V4v (*p*_*adj*_ < 0.0001), between V3 and V3AB (*p*_*adj*_ < 0.0001), V3 and V4v (*p*_*adj*_ = 0.0002), but not between V1 and V2 (*p*_*adj*_ = 0.062) and between V3AB and V4v (*p*_*adj*_ = 0.416).

A series of one-sided one-sample permutation tests (sampling permutation distribution 5000) was performed on the relative BOLD amplitude values of each ROI and test pattern to assess whether these values were greater than zero. The resultant *p* values were corrected with the FDR method for twenty comparisons (i.e. 5 ROIs × 4 test orientations). The results showed that for all the conditions the relative BOLD signal values were significantly greater than zero (*p*_*adj*_ < 0.01). These results indicate reliable adaptation effects on the oriented test GPs.

Altogether, these results show robust responses to oriented test GPs mainly in early visual areas (V1, V2 and V3), but no evidence of orientation-selective adaptation as there was not a significant difference in relative BOLD amplitude between test GP orientations. Moreover, in higher tier extrastriate areas (i.e. V3AB, V4v) the overall amplitude of *HRFs* was further reduced, suggesting a lack of selectivity for oriented GPs.

### Relative percent BOLD signal change

**Figure 2:**
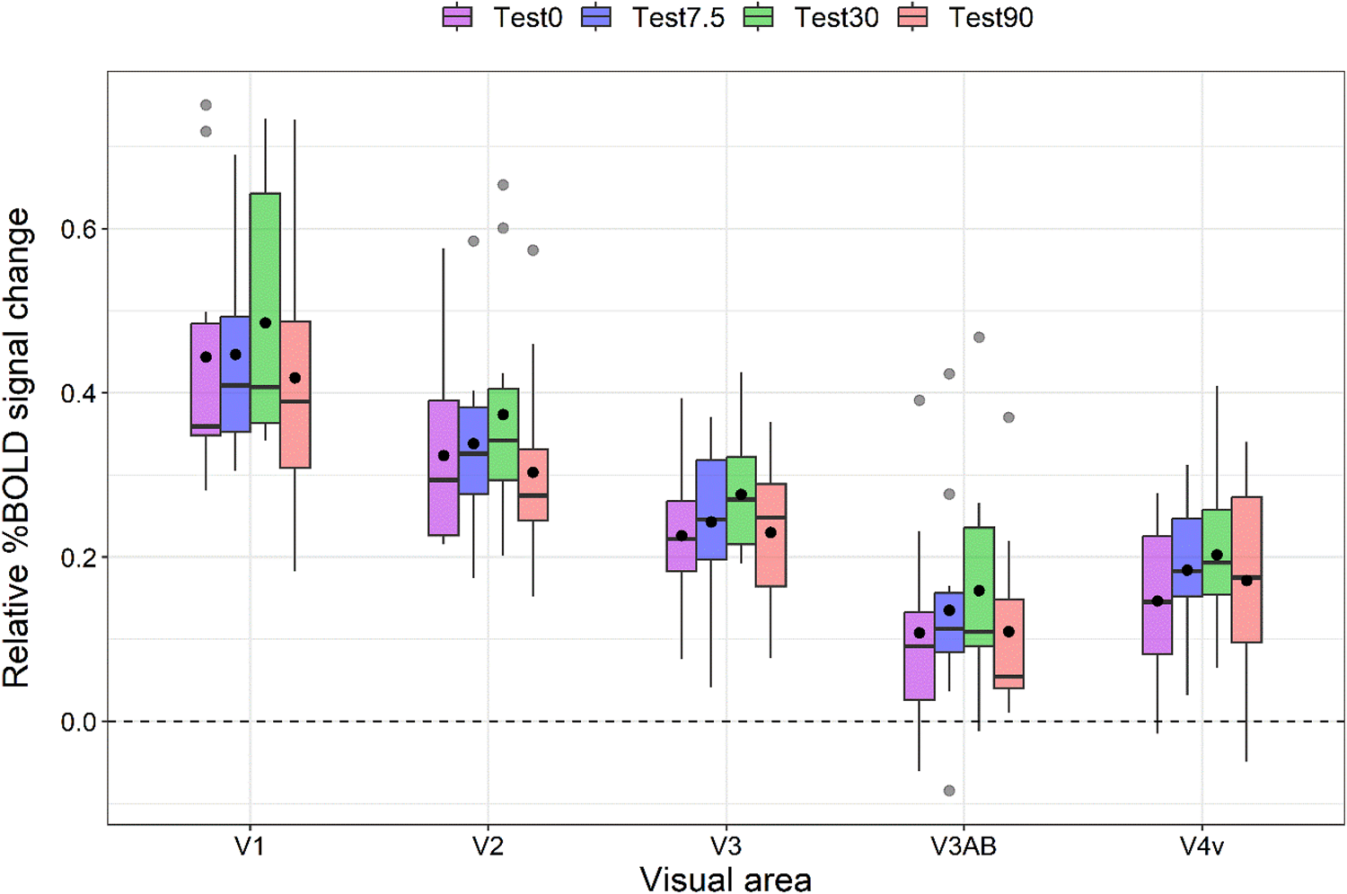
Boxplots of relative percent BOLD signal change for the test and ROI regressors. Each test condition is represented by a distinct color. For each boxplot, the horizontal black line indicates the median and the lower and upper hinges correspond to the first and third quartiles (i.e. the 25th and 75th percentiles). The black point within each boxplot represents the mean response amplitude. The grey points represent outliers.

The relative percent BOLD signal change values for all visual areas and test patterns are reported in Fig. 2. As shown for the *HRF* amplitudes and *HRFs* time courses (Fig. S2 in the Supplementary Material), responses in early visual areas (V1 and V2) were generally stronger than those in higher tier visual areas. To assess for differences in response amplitudes across visual areas and test orientations used, relative percent BOLD signal change values were analyzed with a linear mixed model including as fixed effects the ROI and the test orientation, and as random effect the intercept across participants. According to a Shapiro–Wilk test, the residuals were not normally distributed (*W* = 0.972, *p* = 0.0067), with a moderate skewness of 0.716 (SE: 0.172). Using the median absolute deviation with a cut-off of 3^84^, we also identified 17 outliers that were included in the analysis. Therefore, we used again the Aligned Rank Transform (ART)^85–87^. A linear mixed model with random intercept across participants and including ROI and test orientation as within-subjects factors revealed a significant effect of the ROI (*F*_4, 171_ = 76.21, *p* < 0.0001), a significant effect of test orientation (*F*_3, 171_ = 2.99, *p* = 0.0323), but no significant interaction between ROI and test orientation (*F*_12, 171_ = 0.063, *p* = 0.999). FDR-corrected post hoc comparisons for the ROI (correction for 10 tests) showed a significant difference between all the ROIs: V1 and V2 (*p*_*adj*_ < 0.0001), V1 and V3 (*p*_*adj*_ < 0.0001), V1 and V3AB (*p*_*adj*_ < 0.0001), V1 and V4v (*p*_*adj*_ < 0.0001), V2 and V3 (*p*_*adj*_ < 0.0001), V2 and V3AB (*p*_*adj*_ < 0.0001), V2 and V4v (*p*_*adj*_ < 0.0001), between V3 and V3AB (*p*_*adj*_ < 0.0001), V3 and V4v (*p*_*adj*_ = 0.0002), V3AB and V4v (*p*_*adj*_ = 0.0174). These results suggest greater adaptation effects to vertically oriented GPs mainly in early visual areas (V1, V2, and V3). FDR-corrected post hoc comparisons for the test orientation revealed a significant difference between test at 0° and 30° (*p*_*adj*_ = 0.0432), and between 30° and 90° (*p*_*adj*_ = 0.0221). The other comparisons did not reach statistical significance (*p*_*adj*_ > 0.05).

A series of one-sided one-sample permutation tests (sampling permutation distribution 5000) was performed on the relative BOLD signal values of each ROI and test pattern to assess whether these values were greater than zero. The resultant *p* values were corrected with the FDR method for twenty comparisons (i.e. 5 ROIs × 4 test orientations). The results showed that for all the conditions the relative BOLD signal values were significantly greater than zero (*p*_*adj*_ < 0.05). Again, these results indicate reliable adaptation effects on the oriented test GPs.

Additionally, we examined fMRI contrasts between test orientations and the blank, observing different patterns of activation and deactivation. The outcome of this analysis is reported in the Supplementary Material (see Table S1 and Fig. S4).

### Adaptation index

**Figure 3:**
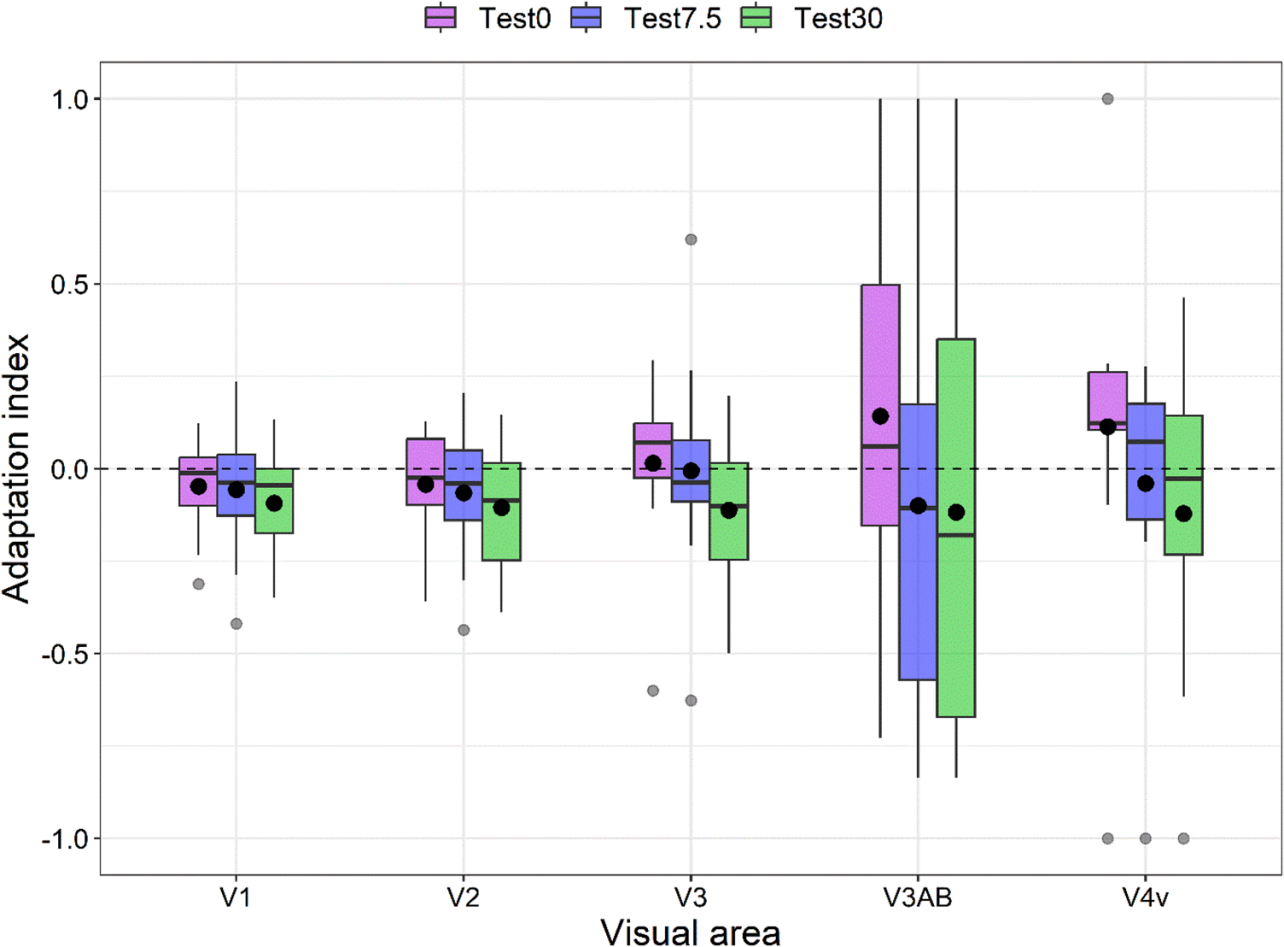
Boxplots of adaptation index values for the test orientations at 0° (parallel), 7.5°, and 30°. Please note that the reason the 90° orientation is not included in the comparison of test orientations is because it was specifically used to calculate the AI. The boxplots of each visual area are grouped together. The black point within each boxplot represents the mean adaptation index, whereas the grey points represent outliers.

Figure 3 shows the adaptation index values for each ROI and test GP orientation. According to a Shapiro–Wilk test, residuals were not normally distributed (*W* = 0.935, *p* < 0.001), with a skewness of 0.087 (SE: 0.198). Using the median absolute deviation with a cut-off of 3, we identified 26 outliers (ranging between − 1 and 1) that were nevertheless included in the analysis. Again, we used the Aligned Rank Transform. A linear mixed model with random intercept across participants and including ROI and test orientation as within-subjects factors revealed only a significant effect of the test orientation (*F*_2, 126_ = 7.32, *p* < 0.001) (ROI: *F*_4, 126_ = 1.62, *p* = 0.173; interaction between ROI and test orientation: *F*_8, 126_ = 0.681, *p* = 0.706). For the main effect of test orientation, FDR-corrected post hoc comparisons revealed a significant difference between the parallel test (i.e. 0°) and the test GP at 7.5° (*p*_*adj*_ = 0.033), between the parallel test and the test GP at 30° (*p*_*adj*_ = 0.0007), but not a significant difference between test GP at 7.5° and 30° (*p*_*adj*_ = 0.141).

A series of one-sided one-sample permutation tests (sampling permutation distribution 5000) were performed on the adaptation indexes (AIs) of each condition to assess whether these values were greater than zero. The resultant *p* values were corrected with the FDR method for fifteen comparisons (i.e. 5 ROIs × 3 test GP orientations). The results showed that none of the AIs were significantly greater than zero (all *p*_*adj*_ > 0.05). These results indicate a lack of orientation-tuned adaptation across the visual areas tested.

## Discussion

Nearly all neural systems exhibit adaptation, which is the sensitivity adjustment in response to a stimulus. Adaptation has also been used as a behavioral method to demonstrate selective neuronal sensitivity to different stimulus dimensions^45^. Based on this assumption, the present study contributes to our understanding of retinotopic visual brain areas that are involved in the processing of form features in oriented GPs using the paradigm of fMRI adaptation^90^. Our findings reveal how the human visual cortex processes oriented visual cues in complex environments. The underlying premise of this paradigm is that if a neuron responds to a specific stimulus configuration, then a second presentation of the same stimulus will elicit a weaker response compared to the first presentation^13^. In the current investigation, following adaptation to vertically oriented GPs, fMRI responses were recorded to test GPs of six different orientations, either 0°, ± 7.5°, ± 30°, or 90° with respect to the standard vertical orientation axis. Specifically, fMRI adaptation allowed us to quantify orientation-selective responses to GPs in the human visual cortex while observers performed a demanding foveal task to control for spatial attention across conditions but also to encourage the maintenance of gaze fixation, which is crucial for maintaining adaptation in neurons with small receptive fields^45,66,91,92^.

Overall, our findings provide neuroimaging evidence showing that the proportion of neurons selective for oriented GPs is greater in early visual areas (V1, V2, and V3) than in higher tier visual areas (V3AB, V4v). Moreover, the initial assumption that significant orientation-tuned adaptation effects would be found in both early visual areas and higher tier visual regions, with greater impact over the extrastriate visual areas, cannot be confirmed. Indeed, the retention of the null hypothesis is supported by the lack of evidence for orientation-selective tuning in both early visual areas and higher tier visual areas (Fig. 2), unlike other stimuli such as Gabors^45^ – probably because GPs are more complex stimuli to visually process than gratings and convey a much weaker orientation signal. Although we used a volume-based ROI analysis, most voxels were aligned along the cortical surface. However, the density of responsive neurons within a given voxel might be less for stimuli like GPs.

In our study, not all GPs test orientations showed the same output: looking at the relative percent BOLD signal change (Fig. 2) we found a significant difference between the parallel test at 0° and the test GP at 30°, and between the test at 30° and the test at 90°, indicating that adaptation to these orientations in GPs may reflect the activity of different orientation-selective neurons, yet no other differences were found indicating that these orientations seem to be encoded similarly after adaptation. The lack of differences between 0° and 90°, i.e. vertical vs. horizontal test GP is surprising as we know from the literature that vertical and horizontal GPs are perceived differently^27^ (see the introduction section for more details on the so-called “horizontal effect”). Different results were obtained by Fang et al.^45^ who performed a study investigating long-term orientation adaptation in the human visual cortex using an event-related fMRI adaptation experiment with oriented Gabor patches. In that study, the fMRI response in V1, V2, V3/VP, V3A, and V4 to the test stimulus after long-term adaptation (i.e. 20 s pre-adaptation and 5 s top-up adaptation) to an oriented pattern was related to the angle difference between the adapting and test stimuli. The Gabor patches, in the test phase, were randomly rotated clockwise or counter-clockwise either 0°, ± 7.5°, ± 30°, or 90° with respect to the adapting Gabor patch as was done for GPs in the current study. The authors found that observers’ threshold changes were influenced by the length of the adaptation phase. Their findings offer compelling fMRI evidence for selective orientation-tuned neurons in human V1 that have been specifically adapted. Further investigations are required to better understand the different neural mechanisms involved in the processing of basic visual stimuli such as oriented GPs, Gabors^45^ and illusory contours^44^.

Although the current study aimed to shed light on the neural bases involved in the processing of vertically oriented GPs, it does not directly address the question of whether our results can be explained in terms of local versus global orientation adaptation. However, it is plausible that local orientation adaptation may have occurred in response to vertically oriented GPs, as a single V1 neuron’s receptive field may cover a few aligned dipoles that resemble a straight- and oriented-line stimulus. This can be a tenable explanation for the findings of the current investigation as local adaptation to vertically oriented GPs can account for the robust adaptation effects in the early visual areas. Nonetheless, a critical aspect of this interpretation is that the receptive fields represent a specific region of the visual field that elicits a response in a particular neuron^93^. As dynamic GPs show a new spatial configuration of the dipoles in each frame of the sequence displayed^27,41,57–60^, it is less probable that the same dipole (or ensemble of dipoles) will adapt a single V1 neuron. Nevertheless, it is probable that different but similar dipoles in terms of local orientation and spatial position may excite and thus adapt the same single V1 neurons. This viewpoint is consistent with single-cell recording studies that have shown that the early visual areas V1 and V2 respond to oriented GPs^5,12^. Indeed, there is physiological evidence in macaque monkeys that simple and complex cells in the early visual areas V1/V2 show selectivity for orientation cues contained in static vertically oriented GPs presented in their classical receptive field^6,12^. Smith et al.^12^ observed that although it is generally known that the cortical visual area V2 of the macaque visual cortex responds to typical oriented grating stimuli, physiological data suggest that this cortical area may also be crucial in the coding of more complex visual stimuli such as GPs. In their study, the authors investigated how V2 cells respond to the form signals in GPs that are restricted to the classical receptive field (CRF). Their findings suggest that V2 neurons behave similarly to V1 neurons in response to the local signals in GPs and that the surrounding global form signal has little impact on these responses. In addition, from the literature, we know that the majority of V1’s cortical output is sent to V2, which then receives a substantial feedback projection in return—both have comparable surface areas and similarly scaled retinotopic maps of the visual field, this explains why they show a similar trend in the processing of vertically oriented GPs (see Sincich and Horton^94^ for a review).

Moreover, in our study, local adaptation may have been produced by using (spatially broadband) dipoles rather than the more conventional oriented Gabors and so the results might have implications for studies that have previously used this type of stimulus and maybe some that use, for example, oriented lines^44^. One explanation might be that dipoles can, in theory, drive two orthogonally oriented cell populations at the same time and so the *net* adaptation might be very small at the population level. In addition, lower contrast energy than Gabor patches in any given spatial band^21,95^ may have produced weaker responses in orientation selective units^5^.

Moving forward, it is crucial for future investigations to delve deeper into the intricate mechanisms underlying orientation-specific adaptation. While our study did not uncover significant findings in terms of neuronal density alone, it highlights the need to explore additional factors that contribute to this complex process. Adaptation is a dynamic and active phenomenon, involving a multitude of interrelated neural circuits and interactions with higher-level cognitive processes. Future research should strive to elucidate the precise nature of these mechanisms, such as the role of feedback connections in shaping orientation-specific adaptation effects. Moreover, employing experimental paradigms such as electroencephalography (EEG) and fMRI may offer valuable insights into the temporal dynamics and network-level mechanisms underlying adaptation^45,96^ (see Lopes da Silva^97^ and Larsson et al.^98^ for a review). By gaining a deeper understanding of the complex interplay between neural activity and adaptation, we can advance our knowledge of visual processing mechanisms.

In conclusion, our findings show evidence of robust effects of adaptation to oriented GPs at the early level of the visual system (V1, V2 and V3) but these effects are less pronounced in higher tier visual areas (V3AB and V4v). In both early and higher tier visual areas the adaptation effects on the BOLD response lack orientation selectivity. From our results, we can infer that form features in oriented GPs are predominantly derived from local processing starting in the primary visual cortex.

## Supplementary Information

Supplementary Information.

## Data Availability

The datasets used and/or analyzed during the current study available from the corresponding author on reasonable request.

## References

[1] Cadieu C (2007). A model of V4 shape selectivity and invariance. J. Neurophysiol..

[2] Desimone R, Schein SJ (1987). Visual properties of neurons in area V4 of the macaque: Sensitivity to stimulus form. J. Neurophysiol..

[3] Maunsell JHR, Newsome WT (1987). Visual processing in monkey extrastriate cortex. Annu. Rev. Neurosci..

[4] Ostwald D, Lam JM, Li S, Kourtzi Z (2008). Neural coding of global form in the human visual cortex. J. Neurophysiol..

[5] Smith MA, Bair W, Movshon JA (2002). Signal in macaque striate cortical neurons that support the perception of glass patterns. J. Neurosci..

[6] Tse PU (2002). Using Glass patterns and fMRI to identify areas that process global form in macaque visual cortex. J. Vis..

[7] Wilson HR, Wilkinson F (1998). Detection of global structure in Glass patterns: Implications for form vision. Vis. Res..

[8] Glass L (1969). Moiré effect from random dots. Nature.

[9] Khuu SK, Moreland A, Phu J (2011). The role of shape-from-shading information in the perception of local and global form in glass patterns. J. Vis..

[10] Mandelli MJF, Kiper DC (2005). The local and global processing of chromatic glass patterns. J. Vis..

[11] Wilson JA, Switkes E, de Valois RL (2004). Glass pattern studies of local and global processing of contrast variations. Vis. Res..

[12] Smith MA, Kohn A, Movshon JA (2007). Glass pattern responses in macaque V2 neurons. J. Vis..

[13] Krekelberg B, Vatakis A, Kourtzi Z (2005). Implied motion from form in the human visual cortex. J. Neurophysiol..

[14] Swettenham JB, Anderson SJ, Thai NJ (2010). MEG responses to the perception of global structure within glass patterns. PLoS One.

[15] Pavan A, Ghin F, Donato R, Campana G, Mather G (2017). The neural basis of form and form-motion integration from static and dynamic translational Glass patterns: A rTMS investigation. Neuroimage.

[16] Glass L, Pérez R (1973). Perception of random dot interference patterns. Nature.

[17] Glass L, Switkes E (1976). Pattern recognition in humans: Correlations which cannot be perceived. Perception.

[18] Clifford CWG, Weston E (2005). Aftereffect of adaptation to Glass patterns. Vis. Res..

[19] Dakin SC (1997). The detection of structure in glass patterns: Psychophysics and computational models. Vis. Res..

[20] Kurki I, Laurinen P, Peromaa T, Saarinen J (2003). Spatial integration in Glass patterns. Perception.

[21] Pavan A, Hocketstaller J, Contillo A, Greenlee MW (2016). Tilt aftereffect following adaptation to translational Glass patterns. Sci. Rep..

[22] Schmidtmann G, Jennings BJ, Bell J, Kingdom FAA (2015). Probability, not linear summation, mediates the detection of concentric orientation-defined textures. J. Vis..

[23] Prazdny K (1986). Some new phenomena in the perception of glass patterns. Biol. Cybern..

[24] Zucker SW (1986). The computational connection in vision: Early orientation selection. Behav. Res. Methods Instrum. Comput..

[25] Wilson HR, Wilkinson F, Asaad W (1997). Concentric orientation summation in human form vision. Vis. Res..

[26] Loffler G, Wilson HR, Wilkinson F (2003). Local and global contributions to shape discrimination. Vis. Res..

[27] Nankoo JF, Madan CR, Spetch ML, Wylie DR (2012). Perception of dynamic Glass patterns. Vis. Res..

[28] Donato R, Pavan A, Campana G (2020). Investigating the interaction between form and motion processing: A review of basic research and clinical evidence. Front. Psychol..

[29] Hansen BC, Essock EA (2004). A horizontal bias in human visual processing orientation and its correspondence to the structural components of natural scenes. J. Vis..

[30] Aspell JE, Wattam-Bell J, Atkinson J, Braddick OJ (2010). Differential human brain activation by vertical and horizontal global visual textures. Exp. Brain Res..

[31] Mannion DJ, Clifford CWG (2011). Cortical and behavioral sensitivity to eccentric polar form. J. Vis..

[32] Mannion DJ, McDonald JS, Clifford CWG (2010). The influence of global form on local orientation anisotropies in human visual cortex. Neuroimage.

[33] Kobatake E, Tanaka K (1994). Neuronal selectivities to complex object features in the ventral visual pathway of the macaque cerebral cortex. J. Neurophysiol..

[34] Pasupathy A, Connor CE (2002). Population coding of shape in area V4. Nat. Neurosci..

[35] Kourtzi Z, Huberle E (2005). Spatiotemporal characteristics of form analysis in the human visual cortex revealed by rapid event-related fMRI adaptation. Neuroimage.

[36] Kourtzi Z, Tolias AS, Altmann CF, Augath M, Logothetis NK (2003). Integration of local features into global shapes: Monkey and human fMRI studies. Neuron.

[37] Altmann CF, Bülthoff HH, Kourtzi Z (2003). Perceptual organization of local elements into global shapes in the human visual cortex. Curr. Biol..

[38] Fitzpatrick D (2000). Seeing beyond the receptive field in primary visual cortex. Curr. Opin. Neurobiol..

[39] Gibson JJ, Radner M (1937). Adaptation, after-effect and contrast in the perception of tilted lines. J. Exp. Psychol..

[40] Jin DZ, Dragoi V, Sur M, Seung HS (2005). Tilt aftereffect and adaptation-induced changes in orientation tuning in visual cortex. J. Neurophysiol..

[41] Pavan A (2021). Spatial and temporal selectivity of translational glass patterns assessed with the tilt after-effect. Iperception.

[42] Larsson J, Landy MS, Heeger DJ (2006). Orientation-selective adaptation to first- and second-order patterns in human visual cortex. J. Neurophysiol..

[43] Sapountzis P, Schluppeck D, Bowtell R, Peirce JW (2010). A comparison of fMRI adaptation and multivariate pattern classification analysis in visual cortex. Neuroimage.

[44] Montaser-Kouhsari L, Landy MS, Heeger DJ, Larsson J (2007). Orientation-selective adaptation to illusory contours in human visual cortex. J. Neurosci..

[45] Fang F, Murray SO, Kersten D, He S (2005). Orientation-tuned fMRI adaptation in human visual cortex. J. Neurophysiol..

[46] Hogendoorn H, Verstraten FAJ (2013). Decoding the motion aftereffect in human visual cortex. Neuroimage.

[47] Huk AC, Ress D, Heeger DJ (2001). Neuronal basis of the motion aftereffect reconsidered. Neuron.

[48] Tonelli A, Pooresmaeili A, Arrighi R (2020). The role of temporal and spatial attention in size adaptation. Front. Neurosci..

[49] Kramer AF, Sirevaag EJ, Hughes PR (1988). Effects of foveal task load on visual-spatial attention: Event-related brain potentials and performance. Psychophysiology.

[50] Bahrami B, Carmel D, Walsh V, Rees G, Lavie N (2008). Spatial attention can modulate unconscious orientation processing. Perception.

[51] Faul F, Erdfelder E, Buchner A, Lang AG (2009). Statistical power analyses using G*Power 3.1: Tests for correlation and regression analyses. Behav. Res. Methods.

[52] Weigelt S, Limbach K, Singer W, Kohler A (2012). Orientation-selective functional magnetic resonance imaging adaptation in primary visual cortex revisited. Hum. Brain Mapp..

[53] World Medical Association declaration of Helsinki (2013). Ethical principles for medical research involving human subjects. JAMA J. Am. Med. Assoc..

[54] Brainard DH (1997). The psychophysics toolbox. Spat. Vis..

[55] Pelli DG (1997). The VideoToolbox software for visual psychophysics: Transforming numbers into movies. Spat. Vis..

[56] Kleiner M (2007). What’s new in Psychtoolbox-3?. Perception.

[57] Pavan A, Bimson LM, Gall MG, Ghin F, Mather G (2017). The interaction between orientation and motion signals in moving oriented Glass patterns. Vis. Neurosci..

[58] Pavan A (2019). Visual short-term memory for coherent motion in video game players: Evidence from a memory-masking paradigm. Sci. Rep..

[59] Ross J (2004). The perceived direction and speed of global motion in Glass pattern sequences. Vis. Res..

[60] Ross J, Badcock DR, Hayes A (2000). Coherent global motion in the absence of coherent velocity signals. Curr. Biol..

[61] Apthorp D, Alais D (2009). Tilt aftereffects and tilt illusions induced by fast translational motion: Evidence for motion streaks. J. Vis..

[62] Clifford CWG, Wenderoth P, Spehar B (2000). A functional angle on some after-effects in cortical vision. Proc. R. Soc. B Biol. Sci..

[63] Gandhi SP, Heeger DJ, Boynton GM (1999). Spatial attention affects brain activity in human primary visual cortex. Proc. Natl. Acad. Sci. U.S.A..

[64] Kastner S, de Weerd P, Ungerleider LG (2000). Texture segregation in the human visual cortex: A functional MRI study. J. Neurophysiol..

[65] Tootell RBH (1998). The retinotopy of visual spatial attention. Neuron.

[66] Somers DC, Dale AM, Seiffert AE, Tootell RBH (1999). Functional MRI reveals spatially specific attentional modulation in human primary visual cortex. Proc. Natl. Acad. Sci. U.S.A..

[67] Chaudhuri A (1990). Modulation of the motion aftereffect by selective attention. Nature.

[68] Lankheet MJM, Verstraten FAJ (1995). Attentional modulation of adaptation to two-component transparent motion. Vis. Res..

[69] Rees G, Frith CD, Lavie N (1997). Modulating irrelevant motion perception by varying attentional load in an unrelated task. Science.

[70] Spivey MJ, Spirn MJ (2000). Selective visual attention modulates the direct tilt aftereffect. Percept. Psychophys..

[71] Moeller S (2010). Multiband multislice GE-EPI at 7 tesla, with 16-fold acceleration using partial parallel imaging with application to high spatial and temporal whole-brain fMRI. Magn. Reson. Med..

[72] Jack CR (2008). The Alzheimer’s disease neuroimaging initiative (ADNI): MRI methods. J. Magn. Reson. Imaging.

[73] Deyoe EA (1996). Mapping striate and extrastriate visual areas in human cerebral cortex. Proc. Natl. Acad. Sci. U.S.A..

[74] Engel SA, Glover GH, Wandell BA (1997). Retinotopic organization in human visual cortex and the spatial precision of functional MRI. Cereb. Cortex.

[75] Sereno MI (1995). Borders of multiple visual areas in humans revealed by functional magnetic resonance imaging. Science.

[76] Frank SM (2020). Attention networks in the parietooccipital cortex modulate activity of the human vestibular cortex during attentive visual processing. J. Neurosci..

[77] Dale AM, Fischl B, Sereno MI (1999). Cortical surface-based analysis: I. Segmentation and surface reconstruction. Neuroimage.

[78] Fischl B, Sereno MI, Dale AM (1999). Cortical surface-based analysis: II. Inflation, flattening, and a surface-based coordinate system. Neuroimage.

[79] Talairach J, Tournoux P (1998). Co-Planar Stereotaxic Atlas of the Human Brain: 3-Dimensional Proportional System: An Approach to Cerebral Imaging.

[80] Friston KJ (1994). Statistical parametric maps in functional imaging: A general linear approach. Hum. Brain Mapp..

[81] Benjamini Y, Hochberg Y (1995). Controlling the false discovery rate: A practical and powerful approach to multiple testing. J. R. Stat. Soc. Ser. B (Methodol.).

[82] Proulx S (2014). Increased sensitivity of fast BOLD fMRI with a subject-specific hemodynamic response function and application to epilepsy. Neuroimage.

[83] Worsley KJ (2002). A general statistical analysis for fMRI data. Neuroimage.

[84] Leys C, Ley C, Klein O, Bernard P, Licata L (2013). Detecting outliers: Do not use standard deviation around the mean, use absolute deviation around the median. J. Exp. Soc. Psychol..

[85] Higgins JJ, Blair RC, Tashtoush S (1990). The aligned rank transform procedure. Conf. Appl. Stat. Agric..

[86] Wobbrock JO, Findlater L, Gergle D, Higgins JJ (2011). The aligned rank transform for nonparametric factorial analyses using only ANOVA procedures. Conf. Hum. Factors Comput. Syst. Proc..

[87] Higgins JJ, Tashtoush S (1994). An aligned rank transform test for interaction. Nonlinear World.

[88] R Core Team *R: A language and environment for statistical computing. R Foundation for Statistical Computing,* 275–286 (2021).

[89] Elkin, L. A., Kay, M., Higgins, J. J. & Wobbrock, J. O. An aligned rank transform procedure for multifactor contrast tests. In *UIST 2021 - Proceedings of the 34th Annual ACM Symposium on User Interface Software and Technology,* 754–768 (2021).

[90] Grill-Spector K, Malach R (2001). fMR-adaptation: A tool for studying the functional properties of human cortical neurons. Acta Psychol. (Amst).

[91] Watanabe J (2002). The human prefrontal and parietal association cortices are involved in NO-GO performances: An event-related fMRI study. Neuroimage.

[92] Brefczynski JA, DeYoe EA (1999). A physiological correlate of the ‘spotlight’ of visual attention. Nat. Neurosci..

[93] Hubel DH, Wiesel TN (1962). Receptive fields, binocular interaction and functional architecture in the cat’s visual cortex. J. Physiol..

[94] Sincich LC, Horton JC (2005). The circuitry of V1 and V2: Integration of color, form, and motion. Annu. Rev. Neurosci..

[95] Carandini M, Heeger DJ, Movshon JA (1997). Linearity and normalization in simple cells of the macaque primary visual cortex. J. Neurosci..

[96] Boynton GM, Finney EM (2003). Orientation-specific adaptation in human visual cortex. J. Neurosci..

[97] Lopes da Silva F (1991). Neural mechanisms underlying brain waves: From neural membranes to networks. Electroencephalogr. Clin. Neurophysiol..

[98] Larsson J, Solomon SG, Kohn A (2016). fMRI adaptation revisited. Cortex.

