# Supplementary material for: Lack of orientation specific adaptation to vertically oriented Glass patterns in human visual cortex: an fMRI adaptation investigation
Source: Sci Rep. 2023 Jul 31;13:12362. doi: 10.1038/s41598-023-39247-7 (PMC10390522; doi:10.1038/s41598-023-39247-7)
Supplement: Supplementary file 1 — Supplementary Information. [file 41598_2023_39247_MOESM1_ESM.docx]

**Lack of orientation specific adaptation to vertically oriented Glass patterns in human visual cortex: an fMRI adaptation investigation**

Andrea Pavan^1,2,3*†^, Wilhelm M. Malloni^2†^, Sebastian M. Frank^2^, Simon Wein^2^, Rita Donato^4,5,6^, Mark W. Greenlee^2^

^1^ University of Bologna, Department of Psychology, Viale Berti Pichat, 5, 40127, Bologna, Italy

^2^ Institute for Experimental Psychology, University of Regensburg, Regensburg 93053, Germany

^3^ School of Psychology, University of Lincoln, Brayford Pool, LN6 7TS Lincoln,

United Kingdom

^4^ Department of General Psychology, University of Padova, Padova, Italy

^5^ Proaction Laboratory, Faculty of Psychology and Educational Sciences, University of Coimbra, Colégio de Jesus, Rua Inácio Duarte 65, 3000-481 Coimbra, Portugal.

^6^ CINEICC, Faculty of Psychology and Educational Sciences, University of Coimbra, Rua Colégio Novo, 3000-115 Coimbra, Portugal.

***Corresponding Author**

Andrea Pavan

University of Bologna

Department of Psychology

Viale Berti Pichat, 5, 40127, Bologna, Italy

Email: [andrea.pavan2@unibo.it](mailto:andrea.pavan2@unibo.it)

^†^Equally contributed authors

**Supplementary Material**

Adaptation stimuli (i.e., panel A: vertical adapting GPs during initial adaptation and panel B: vertical adapting GPs during top-up adaptation) evoked a large positive BOLD response (Figure S1), whereas BOLD responses estimated for the oriented test GPs and the blank condition were characterized by a negative BOLD response (Figure S2), probably because of the long, negative undershoot induced by the top-up. Therefore, after subtracting the BOLD signal change measured in the blank condition to all the test conditions (i.e., 0°, 7.5°, 30°, and 90°) the resulting BOLD signal change was positive (Figure S2, right column).





**Figure S1.** BOLD fitted event time courses (s). Panels **A** and **B** show the fitted BOLD signal change for the vertical adapting GPs and top-up adapting GPs, respectively. Different colors represent the ROIs selected. Shaded areas around the curves represent the standard error of the mean.


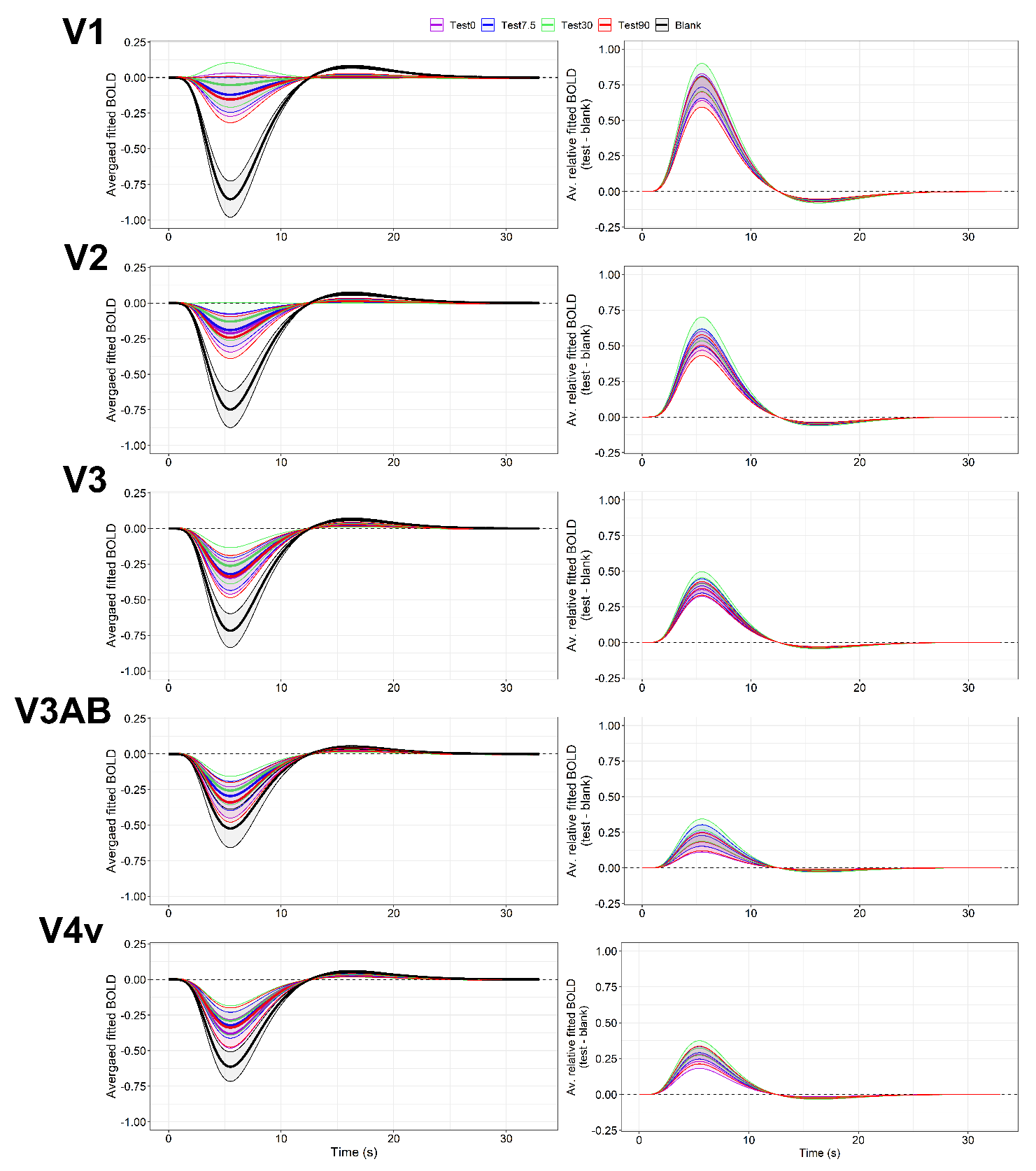


**Figure S2**. The panels in the first column show the BOLD fitted time courses for each test orientation and the blank, separately for each ROI (V1, V2, V3, V3AB, and V4v). Curves represent the BOLD fitted time courses averaged across the ten participants. The BOLD signals change for test orientations is characterized by a strong negative BOLD response across all the ROIs, especially for the blank interval presented after the adaptation period because of the long, negative undershoot induced by the top-up. The panels in the right columns represent the relative BOLD signal change, i.e., when the BOLD estimated for the blank was subtracted from the BOLD of the test patterns. Given the negative sign of the canonical *HRFs*, the relative *HRFs* are now positive. Shaded areas represent the standard error of the mean.

***Analysis of Betas from the canonical HRFs***

For each ROI, beta values were extracted. To assess differences in betas across visual areas and test orientations, beta values were analyzed with a linear mixed model including as fixed effects the ROI and the test orientation, and as random effect the intercept across participants. According to a Shapiro-Wilk test, the residuals were not normally distributed (*W* = 0.945, *p* < 0.0001), with a moderate negative skewness of -0.823 (SE: 0.154). Using the median absolute deviation with a cut-off of 3^1^, we also identified 28 outliers that were included in the analysis. Again, we used the Aligned Rank Transform (ART). A linear mixed model with random intercept across participants and including ROI and test orientation as within-subjects factors, revealed a significant effect of the ROI (*F*_4, 216_ = 5.103, *p* = 0.0006), a significant effect of test orientation (*F*_4, 216_ = 50.163, *p* < 0.0001), and a significant interaction between ROI and test orientation (*F*_16, 216_ = 2.509, *p* = 0.0015). False discovery rate (FDR)^2^-corrected post hoc comparisons for the ROI (correction for 10 tests) showed a significant difference between V1 and V3 (*p_adj_* = 0.01), V1 and V4v (*p_adj_* = 0.0054), V2 and V3 (*p_adj_* = 0.013), and V2 and V4v (*p_adj_* = 0.005), all the other comparisons did not reach significance. For the test orientation, FDR corrected post hoc comparisons (correction for 10 tests) showed a significant difference between test at 0° and 30° (*p_adj_* = 0.02), 0° and blank (*p_adj_* < 0.0001), 7.5° and 30° (*p_adj_* = 0.0457), 7.5° and blank (*p_adj_* < 0.0001), 30° and blank (*p_adj_* < 0.0001), and between 90° and blank (*p_adj_* < 0.0001). All the other comparisons did not reach significance. For the interaction between ROI and test orientation FDR corrected post hoc comparisons (correction for 300 tests) showed, for V1, V2, and V3, a significant difference between all the test orientations and the blank (all *p_adj_* < 0.001), with the blank being significantly lower than the test orientations. However, for V3AB there was a significant difference between all the test orientations and blank (*p_adj_* < 0.05), but not between the test at 0° and blank (*p_adj_* < 0.0937). For area V4v there was also a significant difference between all the test orientations and the blank (all *p_adj_* < 0.05).





**Figure S3.** Boxplots of Beta weights for each ROI and test condition. Each test condition is represented by a distinct grey level, and the boxplots of each ROI are grouped together. For each boxplot, the horizontal black line indicates the median, and the lower and upper hinges correspond to the first and third quartiles (i.e., the 25th and 75th percentiles). The black point within each boxplot represents the mean response amplitude. The grey points represent outliers.

We also examined fMRI contrasts between test orientations and between test orientations and the blank, observing different patterns of activation and deactivation. Table S1 reports the contrasts, number of voxels identified, t-value, MNI-coordinates, and the relative area. Figure S4 shows the same contrasts as Table S1 on the cortical surface.

| Contrast | Number of voxels | t-value | MNI-coordinates (x, y, z) | Area |
| --- | --- | --- | --- | --- |
| 0° > 30° | 16 | -5.54 | 58, -14, 14 | Right Supramarginal Gyrus (BA40) |
|  | 13 | -6.43 | 26, -72, -8 | Right Visual Associative (BA19) |
| 0° > 90° | 37 | -7.54 | 52, 10, 18 | Right Broca Operculus (BA44) |
|  | 16 | -7.32 | 58, 24, 6 | Right Broca Triangle (BA45) |
| 0° > blank | 568 | 9.81 | -18, -94, 2 | Left Secondary visual area (BA18) |
|  | 130 | 7.16 | 20, -84, -6 | Right Secondary visual area (BA18) |
|  | 80 | -7.90 | -4, -8, 66 | Left Premotor and Supplementary Motor (BA6) |
|  | 71 | -9.13 | 52, -30, 36 | Right Supramarginal gyrus (BA40) |
|  | 27 | -6.65 | -50, -30, 42 | Left Supramarginal gyrus (BA40) |
|  | 25 | -5.86 | -50, -46, 24 | Right Angular gyrus (BA39) |
|  | 23 | -6.46 | 56, 2, -10 | Right Superior Temporal gyrus (BA22) |
|  | 21 | -7.30 | 60, -34, 28 | Right Supramarginal gyrus (BA40) |
|  | 21 | -6.05 | 30, -54, 48 | Right Parietal Inferior (BA7) |
| 7.5° > blank | 1968 | 15.20 | 20, -96, 8 | Right Secondary visual area (BA18) |
|  | 15 | 6.88 | 28, -76, 16 | Right Visual Associative (BA19) |
| 30° > 90° | 24 | -7.49 | 58, 24, 8 | Right Broca Triangle (BA45) |
|  | 12 | -5.31 | -54, 16, 18 | Left Broca Operculus (BA44) |
| 30° > blank | 1415 | 10.35 | -22, -84, -2 | Left and Right Secondary visual area (BA18) |
|  | 11 | 5.01 | -22, -68, -4 | Left Visual associative area (BA19) |
|  | 33 | -6.67 | -62, -46, 32 | Left Angular gyrus (BA39) |
|  | 27 | -6.69 | -54, 18, 18 | Left Broca Operculus (BA44) |
|  | 25 | -8.94 | 56, 22, 14 | Right Broca Operculus (BA44) |
|  | 12 | -5.48 | -36, 6, 30 | Left Frontal Eye Fields (BA8) |
|  | 12 | -8.74 | 44, -74, 36 | Right Angular gyrus (BA39) |
| 90° > blank | 437 | 7.22 | -4, -82, -6 | Left Secondary visual area (BA18) |
|  | 156 | 9.11 | 6, -94, 10 | Right Secondary visual area (BA18) |
|  | 114 | 7.52 | -20, -92, 6 | Left Secondary visual area (BA18) |
|  | 35 | -8.65 | 16, -58, 28 | Right Ventral posterior cingulate cortex (BA23) |

**Table S1.** Number of voxels, t-value, and MNI-coordinates of the contrasts between the different test orientations used and between test orientations and blank. Areas were sorted by size of activations (number of voxels) and by *t-value* (positive and negative). Brodmann areas are abbreviated in parentheses (e.g., BA1).


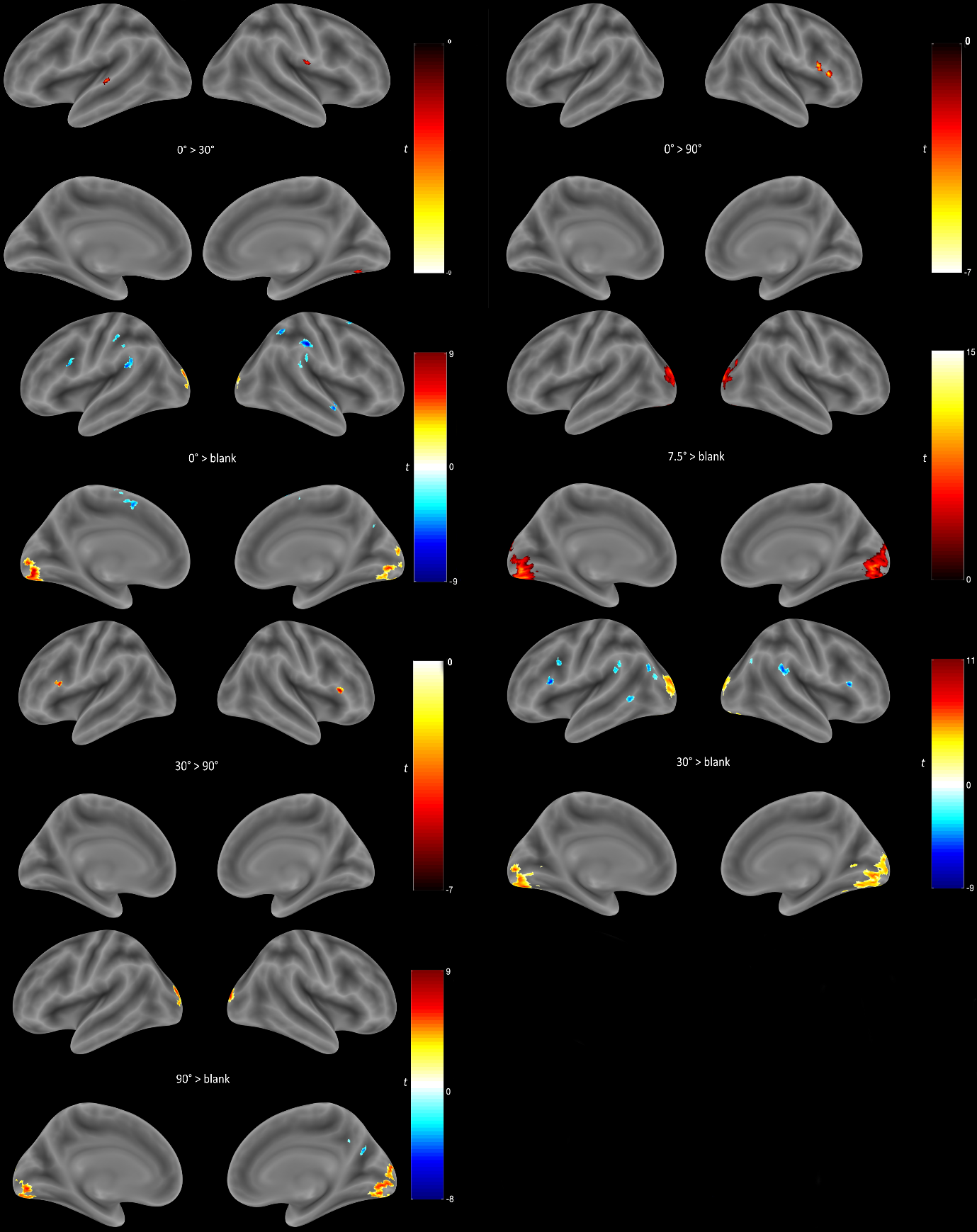


**Figure S4.** Representation of the contrasts reported in Table S1 on the cortical surface. The type of contrast is reported in the middle of each panel (e.g., 0^o^ > blank) and the sidebars indicate t-values; warm colors indicate activation patterns, whereas cold colors deactivation patterns.

**References**

1. Leys, C., Ley, C., Klein, O., Bernard, P. & Licata, L. Detecting outliers: Do not use standard deviation around the mean, use absolute deviation around the median. *J Exp Soc Psychol* **49**, (2013).

2. Benjamini, Y. & Hochberg, Y. Controlling the False Discovery Rate: A Practical and Powerful Approach to Multiple Testing. *Journal of the Royal Statistical Society: Series B (Methodological)* **57**, (1995).
